# A simple classification system (the *Tree* flowchart*)* for breast MRI can reduce the number of unnecessary biopsies in MRI-only lesions

**DOI:** 10.1007/s00330-017-4755-6

**Published:** 2017-03-08

**Authors:** Ramona Woitek, Claudio Spick, Melanie Schernthaner, Margaretha Rudas, Panagiotis Kapetas, Maria Bernathova, Julia Furtner, Katja Pinker, Thomas H. Helbich, Pascal A. T. Baltzer

**Affiliations:** 1 Department of Biomedical Imaging and Image-Guided Therapy, Medical University of Vienna, Vienna, Austria; 2 Clinical Institute of Pathology, Medical University of Vienna, Vienna, Austria

**Keywords:** Magnetic resonance imaging, Breast cancer, Scoring system, Image-guided biopsy, ROC curve

## Abstract

**Objectives:**

To assess whether using the *Tree* flowchart obviates unnecessary magnetic resonance imaging (MRI)-guided biopsies in breast lesions only visible on MRI.

**Methods:**

This retrospective IRB-approved study evaluated consecutive suspicious (BI-RADS 4) breast lesions only visible on MRI that were referred to our institution for MRI-guided biopsy. All lesions were evaluated according to the *Tree* flowchart for breast MRI by experienced readers. The *Tree* flowchart is a decision rule that assigns levels of suspicion to specific combinations of diagnostic criteria. Receiver operating characteristic (ROC) curve analysis was used to evaluate diagnostic accuracy. To assess reproducibility by kappa statistics, a second reader rated a subset of 82 patients.

**Results:**

There were 454 patients with 469 histopathologically verified lesions included (98 malignant, 371 benign lesions). The area under the curve (AUC) of the *Tree* flowchart was 0.873 (95% CI: 0.839–0.901). The inter-reader agreement was almost perfect (kappa: 0.944; 95% CI 0.889–0.998). ROC analysis revealed exclusively benign lesions if the *Tree* node was ≤2, potentially avoiding unnecessary biopsies in 103 cases (27.8%).

**Conclusions:**

Using the *Tree* flowchart in breast lesions only visible on MRI, more than 25% of biopsies could be avoided without missing any breast cancer.

***Key Points*:**

• *The Tree* *flowchart may obviate >25% of unnecessary MRI-guided breast biopsies.*

*• This decrease in MRI-guided biopsies does not cause any false-negative cases.*

• *The Tree* *flowchart predicts 30.6% of malignancies with >98% specificity.*

• *The Tree’s* *high specificity aids in decision-making after benign biopsy results.*

**Electronic supplementary material:**

The online version of this article (doi:10.1007/s00330-017-4755-6) contains supplementary material, which is available to authorized users.

## Introduction

Breast lesions rated as suspicious for cancer according to the American College of Radiology Breast Imaging and Reporting Data System (ACR BI-RADS) (e.g. assigned an ACR BI-RADS ≥ 4 category) that were detected on breast magnetic resonance imaging (MRI) require tissue sampling and histopathological workup [1–3]. Unless these lesions are visible on other imaging modalities, they require dedicated MRI-guided, vacuum-assisted breast biopsy (VABB) to provide representative tissue sampling [1, 2, 4]. As reflected by positive predictive values of MRI-guided biopsies below 50% in the literature [5–8], a relevant number of benign lesions visible on MRI undergo unnecessary VABBs that can potentially be avoided. MR-guided VABB is a safe and accurate procedure in the diagnostic workup, but its application is limited by availability, relatively high costs compared to other biopsy techniques, and the necessity to administer gadolinium-containing contrast agent intravenously [9]. Although minimally invasive, MR-guided biopsies carry a low risk for complications, such as infection or bleeding [9]. There is general consent that unnecessary biopsies should be avoided [10] by ruling out malignancy based on imaging features. Numerous efforts have been made to decrease the number of false-positive results in standard breast MRI using additional imaging techniques, such as diffusion-weighted imaging (DWI), MR spectroscopy (MRS) and positron emission tomography (PET), or sophisticated evaluation of dynamic contrast-enhanced MRI [11–17]. Although these approaches may increase specificity, there are several issues regarding standardization, as well as time and cost effectiveness. It would be thus desirable if such an increase in specificity could be achieved using standard breast MRI sequences only.

To report imaging features and indicate suspicion for malignancy, ACR BI-RADS is the most widely used standard. It facilitates communication among physicians with its structured common language and standardized terminology for image interpretation and reporting. However, it lacks precise rules according to which to assign imaging features (i.e. lesion morphology and functional contrast enhancement kinetics) to diagnostic categories. Therefore, using ACR BI-RADS, the inter-reader agreement remains moderate, diagnostic accuracy is variable, a relevant number of unnecessary biopsies are performed in benign lesions, the inter-reader agreement remains moderate, and diagnostic accuracy is variable [18–22].

**Fig. 1:**
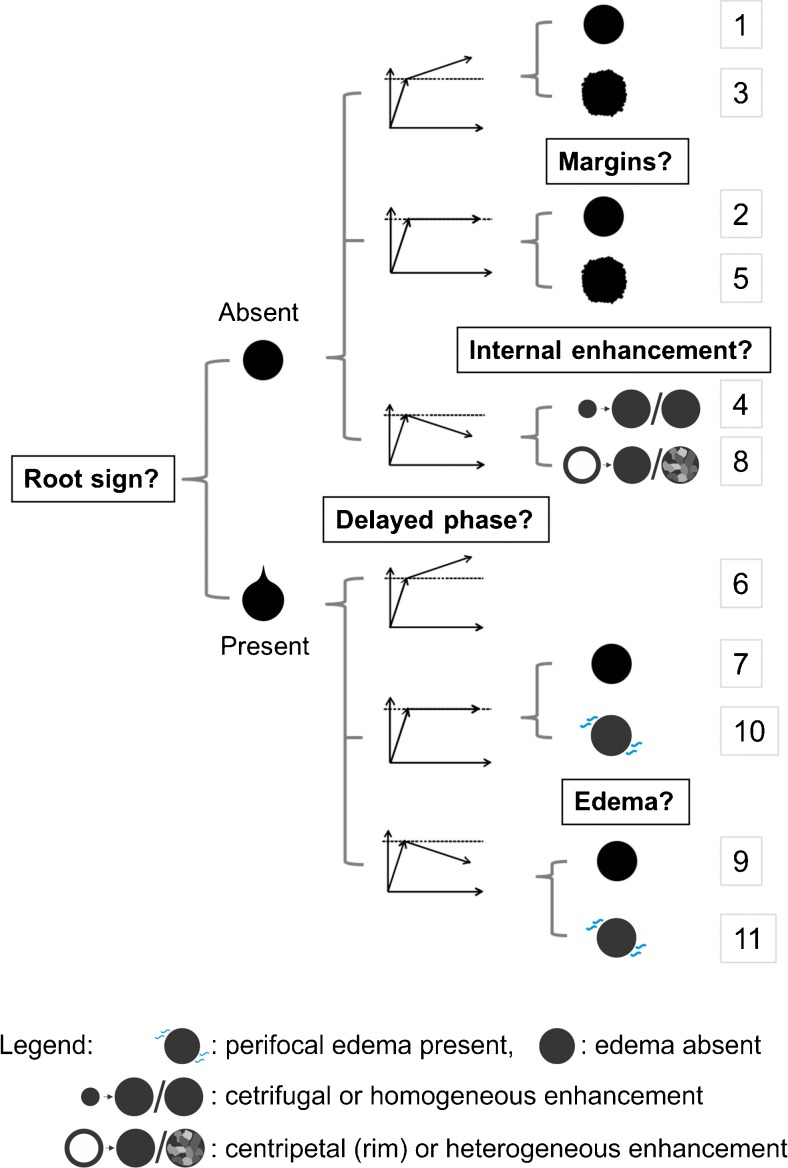
*Tree* flowchart following the description by Marino et al. [24]. Terminal nodes are hierarchically ordered (1–11) and represent increasing probabilities of malignancy

To complement the ACR BI-RADS lexicon and to increase specificity, Baltzer et al. proposed a simple classification system (the *Tree* flowchart*)* for breast MRI to differentiate benign and malignant lesions on breast MRI [23].The *Tree* flowchart combines five diagnostic criteria (the root sign, enhancement kinetics, lesion margins, internal enhancement pattern and ipsilateral oedema; Fig. 1, Table 1) to assign a diagnostic score to each lesion, indicating the likelihood of malignancy. These five criteria were selected from a larger pool of 17 criteria, based on their representation of possibly malignant lesion features [25–28] and their non-redundancy [23]. Neither the initial exploratory evaluation of the *Tree* flowchart [23] nor the subsequent independent validation study [24] specifically addressed a defined clinical setting where the *Tree* could improve clinical management.

**Table 1:**
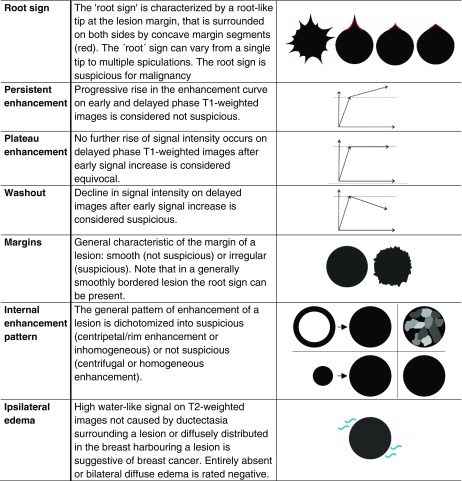
The morphological and kinetic criteria included in the *Tree* flowchart

Thus, the aim of this study was to assess whether using the *Tree* flowchart obviates unnecessary MRI-guided biopsies in MRI-only breast lesions.

## Materials and methods

### Study design

This cross-sectional, retrospective, single-centre study was approved by our institutional review board (IRB). The necessity for informed consent was waived. There were 454 consecutive patients (mean age 52±13 years) with 469 breast lesions visible only on MRI undergoing MRI-guided VABB and/or surgical biopsy at our institution from January 2006 to December 2013 who were included. There was a partial overlap of the data analysed in this study with two prior publications [11, 22]. However, study rationale and results differed between the studies.

#### Imaging and MRI-guided biopsies

Based on our ethical review board-approved study protocol, a database was populated with the results obtained during retrospective readings of diagnostic breast MRI scans. MRI scans were performed in accordance with the EUSOMA (European Society of Breast Cancer Specialists) recommendations [2] at different referring institutions, on 1.5- and 3-Tesla (T) units of different vendors, using dedicated breast coils: seven patients (0.2%) were scanned at 1.0-T, 422 patients (93%) at 1.5-T and 25 patients (5.5%) at 3.0-T. There were 383 examinations (84.4%) acquired using Siemens MAGNETOM scanners (Erlangen, Germany): Avanto (261 patients, 57.5%), Symphony (50 patients, 11%), Essenza (21 patients, 4.6%), Espree (18 patients, 4%), Trio (17 patients, 37.4%), Harmony (seven patients, 1.5%), Verio (three patients, 0.7%), Aera (three patients, 0.7%), Skyra (one patient, 0.2%) and Vision (one patient, 0.2%). Seventy-one examinations (15.6%) were acquired on Philips Scanners (Philips Medical Systems, Best, The Netherlands): Intera (61 patients, 13.4%), Achieva (seven patients, 1.5%) and Ingenia (four patients, 0.9%). The following contrast agents were applied intravenously at dosages of 0.1 mmol gadolinium/kg body weight: Dotarem (gadoterate meglumine/Guerbet, Villepinte, France), Multihance (gadobenate dimeglumine/Bracco Diagnostics, Princeton, NJ, USA), Gadovist (gadobutrol/Bayer Pharmaceuticals, Berlin, Germany), Prohance (gadoteridol/Bracco Diagnostics, Princeton, NJ, USA) and Omniscan (gadodiamide/ GE Healthcare, Princeton, NJ, USA). The detailed sequence parameters of the T2-weighted and the dynamic T1-weighted sequences are shown in ESM 1.

All lesions classified as BI-RADS 4 (suspicious) were biopsied under MRI-guidance at our institution on a 1.5-T system (Avanto, Siemens) using a dedicated double breast imaging and intervention coil (InVivo, Philips).

Written informed consent for the MRI-guided VABB was obtained from all patients in advance.

MRI-guided VABBs were performed as previously described [22]. For lesion localization, a shortened imaging protocol was acquired: before and after the application of intravenous (IV) contrast agent, dynamic, contrast-enhanced, T1-weighted gradient echo sequences were acquired and subtraction images were obtained. The T1-weighted sequence was repeated to verify needle positioning during the biopsy. After VABB, the biopsy sites were marked with MRI-compatible radiopaque clip markers. An experienced board-certified breast pathologist (M.R.) performed histopathological tissue analyses and applied the B classification for diagnosis [29]. Histopathological results and the MRI were compared in interdisciplinary consensus. In cases of discrepancy between imaging results and histopathology, and in lesions with uncertain malignant potential (B3), surgical biopsy was performed, after wire localization, by board-certified, experienced breast surgeons. In case of a benign finding at histopathology, the patients were followed up with breast MRI for at least 12 months.

### Data analysis

All examinations were analysed by an experienced breast imaging radiologist (P.A.B, >10 years of breast MRI experience), and 82 consecutive cases were read by a second reader independently (C.S., radiology resident) to assess inter-reader agreement. Both readers were blinded to the final histopathological diagnosis and previous reading results, if available.

**Fig. 2:**
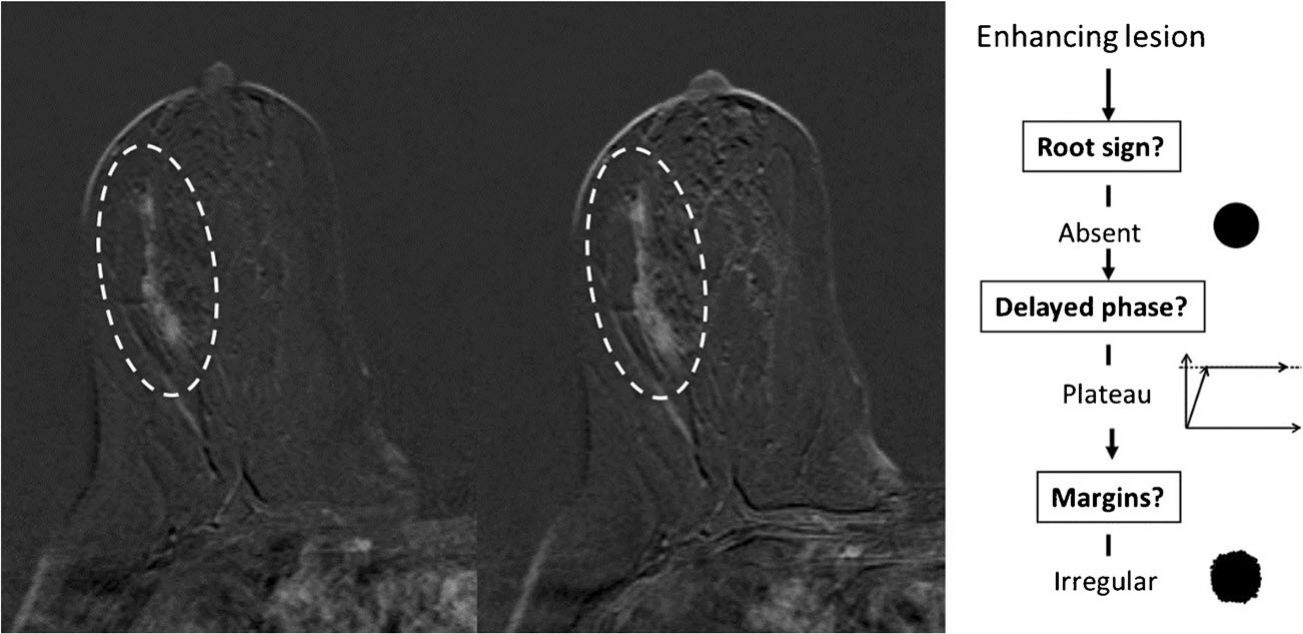
Example of a non-mass lesion: ductal carcinoma in situ (DCIS) Grade 3, presenting as a non-mass lesion without the root sign, with plateau enhancement during the delayed phase and with irregular margins. Based on the *Tree* flowchart (Fig. 1), the described characteristics resulted in a node 5 rating where malignancy cannot be ruled out

**Fig. 3:**
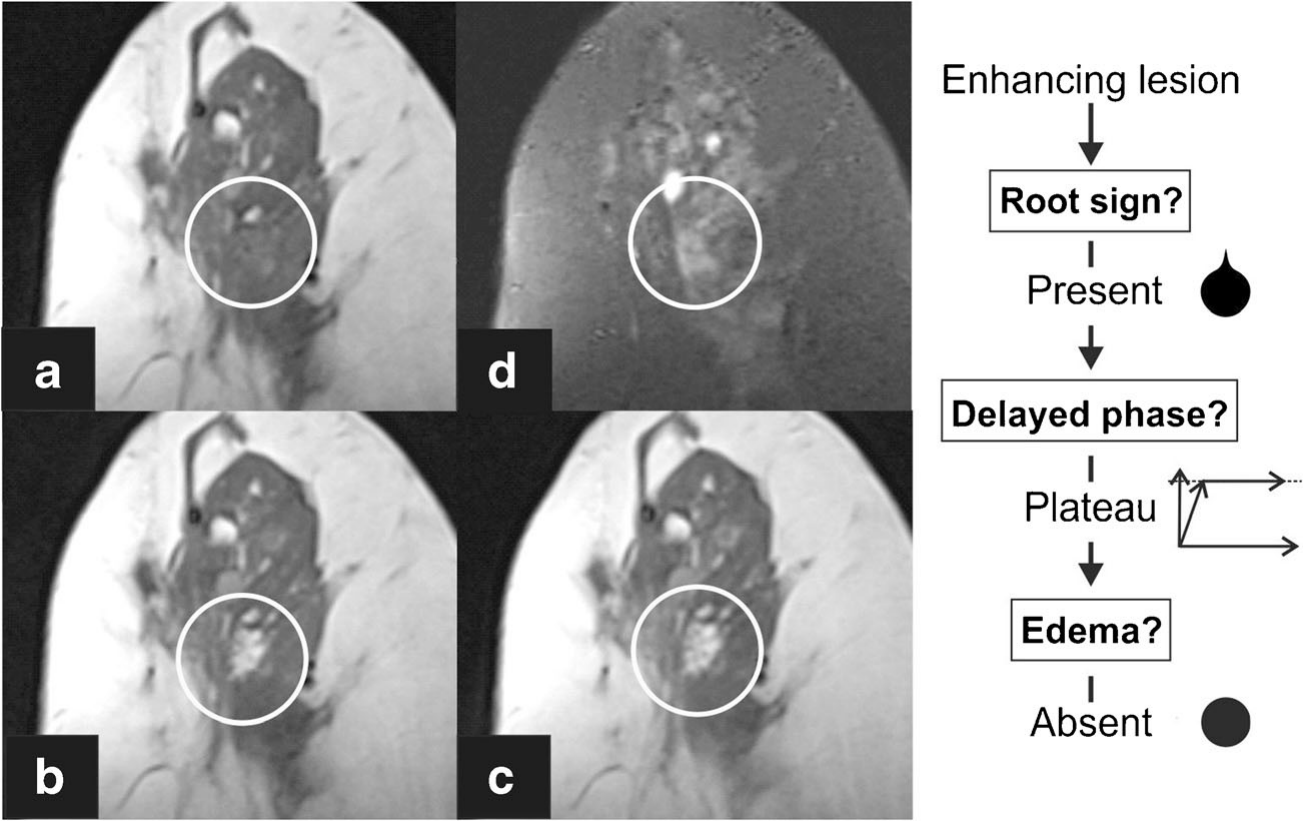
Apocrine ductal carcinoma in situ (DCIS) Grade 2 presenting as a mass lesion with the root sign, delayed plateau enhancement and perifocal oedema, resulting in a classification as *Tree* node 10. Representative axial slices of the T1-weighted, non-enhanced sequence (**a**), a T2 TIRM sequence (**b**), early (**c**) and delayed (**d**) post-contrast T1-weighted sequences are shown

**Fig. 4:**
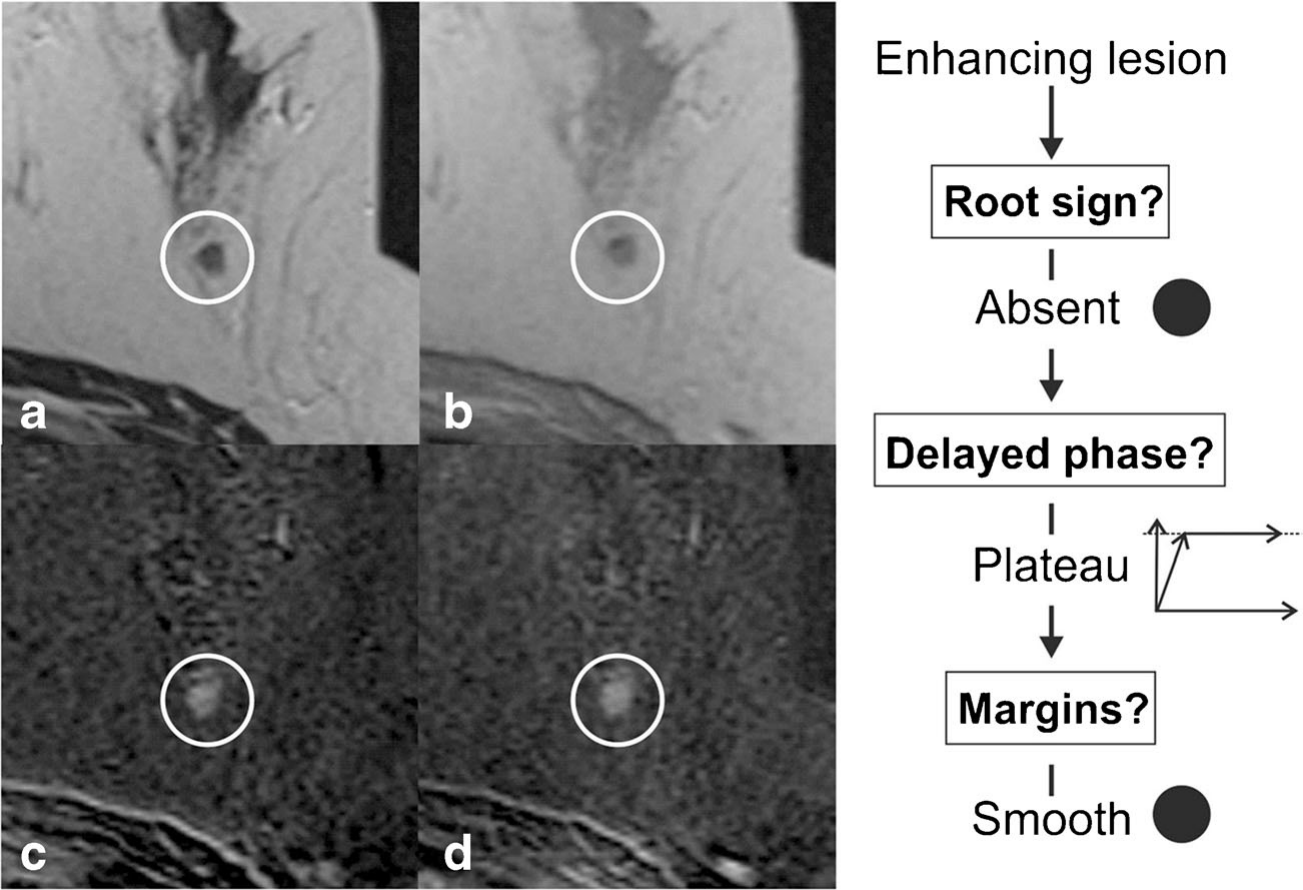
Benign columnar cell change and flat epithelial hyperplasia were diagnosed in this mass lesion showing plateau enhancement and smooth margins without the root sign, thus resulting in a *Tree* node 2. Biopsy could have been avoided with the *Tree* classification in this lesion. Representative slices of the T2-weighted (**a**) and the unenhanced T1-weighted sequences (**b**) are shown, as well as early (**c**) and delayed (**d**) subtractions of contrast-enhanced T1-weighted sequences

**Fig. 5:**
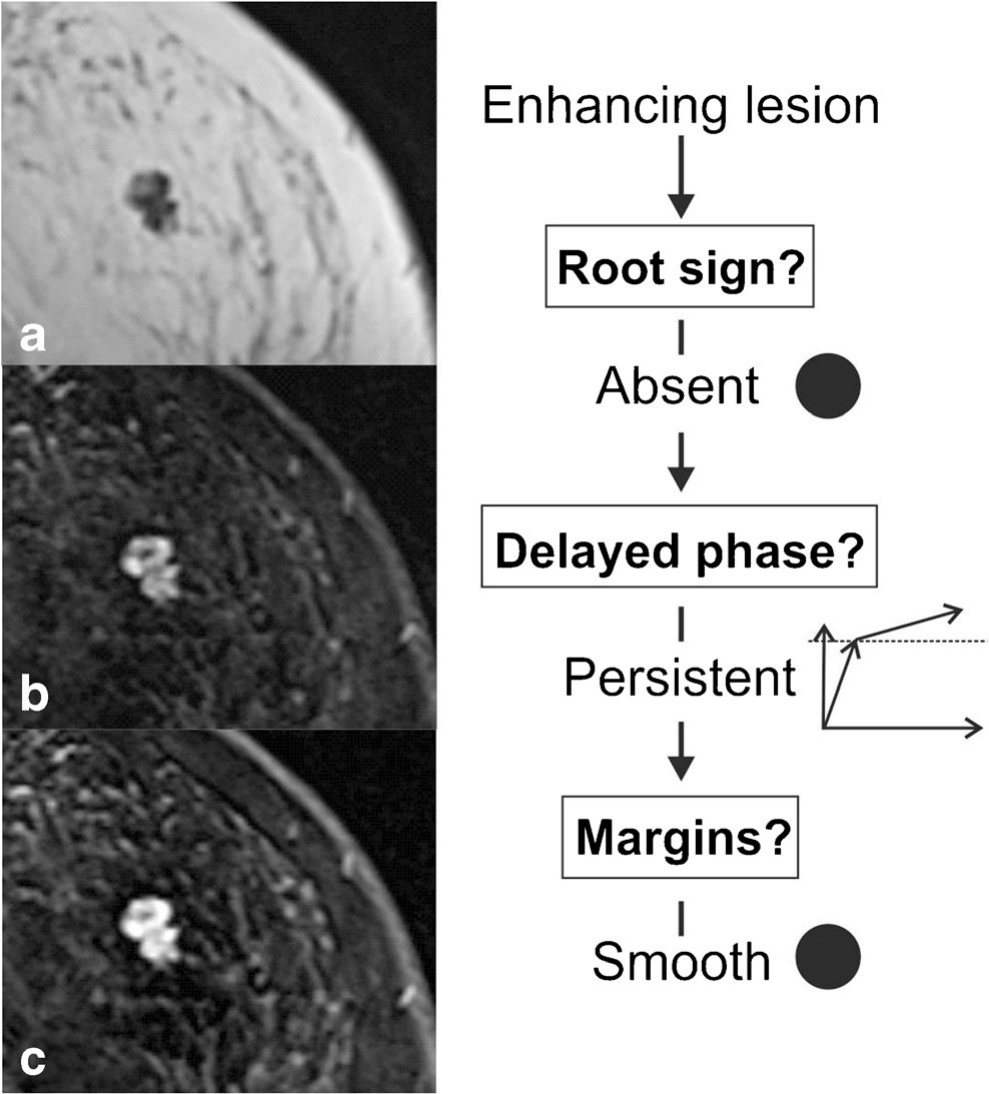
Fibroadenomatous hyperplasia presenting as a mass lesion with persistent enhancement and smooth borders without the root sign (note the lobulations with konvex tips that do not fulfill the criteria of the root sign). The lesion was thus classified as node 1 and could have been identified as benign using the *Tree* flowchart. Representative slices of a non-enhanced T1-weighted sequence (**a**) and subtractions of the early (**b**) and delayed (**c**) post-contrast T1-weighted sequences are shown

The readers were asked to classify all identified lesions following the *Tree* flowchart. This simple classification system is based on five morphological and kinetic criteria (root sign, contrast enhancement kinetics, lesion margins, internal enhancement patterns and oedema; Table 1 and Fig. 1) evaluated on T2-weighted sequences and dynamic, contrast-enhanced, T1-weighted sequences. The *Tree* flowchart contains 11 assignment categories that correspond to an increasing probability of malignancy (1 = lowest, cancer very unlikely, to 11 = highest, cancer very likely; Fig. 1) [23, 24]. Examples of lesions are given in Figs. 2, 3, 4 and 5. A diagnostic category was chosen for each lesion by following the *Tree* flowchart and was noted in a spreadsheet (Fig. 1).

### Statistical analyses

SPSS 23.0 (SPSS, IBM, Chicago, IL, USA) and MedCalc 15 (MedCalc software bvba, Ostend, Belgium) were used for statistical analyses. Inter-reader agreement in the assigned *Tree* categories was assessed using kappa statistics. A receiver operating characteristic (ROC) analysis was performed and the area under the ROC curve was measured to determine overall diagnostic performance. Sensitivity, specificity and likelihood ratios were calculated at different cut-off values. P-values ≤0.05 were considered statistically significant. Two cut-off values, to rule out and rule in malignancy, were selected according to the calculated sensitivities, specificities and numbers of false-positive and false-negative results (low cut-off value with highest sensitivity and lowest false-positive results; high cut-off value with high specificity and low false-negative results).

## Results

### Patients and lesions

Ninety-eight of the included 469 breast lesions (20.9%) were histopathologically diagnosed as malignant, and 371 (79.1%) lesions as benign.

There were 270 lesions (57.6%) that presented as masses, whereas 199 (42.4%) were non-mass lesions. Of all 270 mass lesions, 68 (25.2%) were malignant and 202 (74.8%) benign. Of the 199 non-mass lesions, 30 (15.1%) were malignant and 169 (84.9%) benign. Detailed size descriptions and descriptive statistics of histopathological diagnoses are given in Tables 2 and 3.

**Table 2:** Size distributions in mass and non-mass lesions stratified by histopathological results

Lesion type	n	Mean diameter (mm)	Histology	n	Mean diameter (mm)
Mass lesions	270	9±5 (SD)	Malignant	68	11±6 (SD)
			Benign	202	9±5 (SD)
Non-mass lesions	199	26±15 (SD)	Malignant	30	35±16 (SD)
			Benign	169	24±14 (SD)
Total	469	16±13 (SD)	Malignant	98	18±15 (SD)
			Benign	371	16±12 (SD)

*SD* standard deviation, *n* number

**Table 3:** Descriptive statistics for histopathological diagnoses among all lesions stratified by *Tree* nodes

**Node**		Benign	High-risk	DCIS	Invasive cancer	Total
**1**	n	69	8	0	0	77
	%	*89.6*	*10.4*	*0.0*	*0.0*	
**2**	n	23	3	0	0	26
	%	*88.5*	*11.5*	*0.0*	*0.0*	
**3**	n	154	45	9	9	209
	%	*73.7*	*21.5*	*4.3*	*4.3*	
**4**	n	11	1	0	1	13
	%	*84.6*	*7.7*	*0.0*	*7.7*	
**5**	n	31	9	10	8	58
	%	*53.4*	*15.5*	*17.2*	*13.8*	
**7**	n	10	4	5	9	28
	%	*35.7*	*14.3*	*17.9*	*32.1*	
**8**	n	10	6	6	2	24
	%	*41.7*	*25.0*	*25.0*	*8.3*	
**9**	n	4	1	1	11	17
	%	*23.5*	*5.9*	*5.9*	*64.7*	
**10**	n	3	0	2	5	10
	%	*30.0*	*0.0*	*20.0*	*50.0*	
**11**	n	1	0	0	6	7
	%	*14.3*	*0.0*	*0.0*	*85.7*	
∑	**n**	**316**	**77**	**31**	**45**	**469**
	%	***67*** *.* ***4***	***16*** *.* ***4***	***6*** *.* ***6***	***9*** *.* ***6***	***100*** *.* ***0***

*n* number, ductal carcinoma in situ, *High-risk* high-risk lesion

### ROC curve analyses

**Fig. 6:**
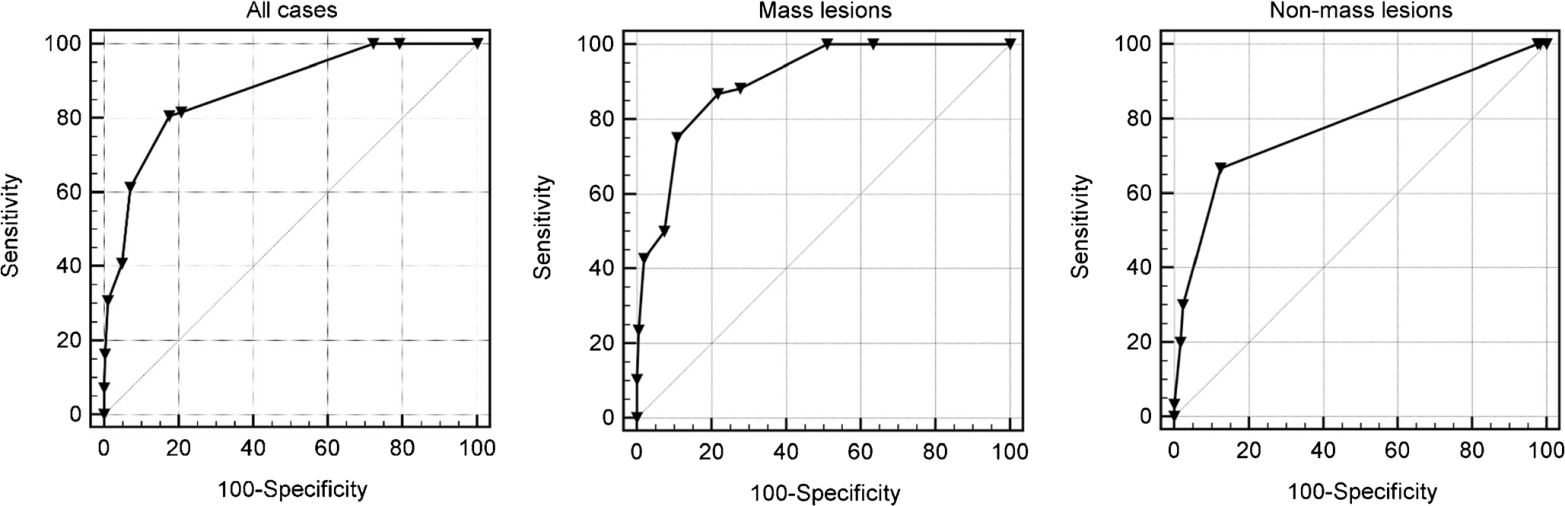
Receiver operating characteristic (ROC) curves of all lesions included in the study, mass lesions and non-mass lesions. Details on the area under the curve (AUC) are given in the text and on diagnostic cut-off values in Tables 4, 5 and 6

Using the *Tree* flowchart for all lesions, the overall accuracy represented by the area under the ROC curve (AUC) was 0.873 (95% CI: 0.839–0.901; *P*<0.0001) (Fig. 6). Detailed results using the *Tree* flowchart in all lesions, masses or non-mass lesions, and details of different cut-off levels and their diagnostic parameters, are shown in Tables 4, 5 and 6. For mass lesions, the AUC for lesion diagnosis was 0.902 (95% CI: 0.860–0.935; *P*<0.0001), whereas in non-mass lesions the AUC was 0.786 (95% CI: 0.722–0.841; *P*<0.0001) (Fig. 6). MRI units by two vendors were used in this study: Siemens (n = 383) and Philips (n = 71) (ESM 1). The diagnostic performance using the *Tree* algorithm did not differ between cases examined on either vendor’s scanners (AUC Siemens = 0.880, standard error = 0.019; AUC Philips = 0.859, standard error = 0.056; *P*=0.724).

**Table 4:** Diagnostic parameters and cut-off values of the *Tree* flowchart in all lesions included in the study

All lesions						
Cut-off	Sens	95% CI	Spec	95% CI	+LR	−LR
≥1	100% (98/98)	96.3–100	0% (0/371)	0–1	1	
>2	100% (98/98)	96.3–100	27.8% (103/371)	23.3–32.6	1.38	0
>3	81.6% (80/98)	72.5–88.7	79.3% (294/371)	74.8–83.3	3.93	0.23
>4	80.6% (79/98)	71.4–87.9	82.5% (306/371)	78.2–86.2	4.6	0.24
>5	61.2% (60/98)	50.8–70.9	93% (345/371)	89.9–95.4	8.74	0.42
>7	40.8% (40/98)	31.0–51.2	95.2% (353/371)	92.4–97.1	8.41	0.62
>8	30.6% (30/98)	21.7–40.7	99% (367/371)	97.3–99.7	28.39	0.7
>9	16.3% (16/98)	9.6–25.2	99.7% (370/371)	98.5–100	60.57	0.84
>10	7.1% (7/98)	2.9–14.2	100% (371/371)	99–100		0.93
>11	0% (0/98)	0–3.7	100% (371/371)	99–100		1

*n* number, *Ben* benign, *Mal* malignant, *Sens* sensitivity, *CI* confidence interval, *Spec* specificity, *+LR* positive likelihood ratio, −LR negative likelihood ratio, *∑* sum

**Table 5:** Diagnostic parameters and cut-off values of the *Tree* flowchart in mass lesions

Mass lesions						
Cut-off	Sens	95% CI	Spec	95% CI	+LR	−LR
≥1	100% (68/68)	94. 7–100	0% (0/202)	0–1.8	1	
>2	100% (68/68)	94.7–100	49% (99/202)	41.9–56.1	1.96	0
>3	88.2% (60/68)	78.1–94.8	72.3% (146/202)	65.6–78.3	3.18	0.16
>4	86.8% (59/68)	76.4–93.8	78.2% (158/202)	71.9–83.7	3.98	0.17
>5	75% (51/68)	63–84.7	89.1% (180/202)	84–93	6.89	0.28
>7	50% (34/68)	37.6–62.4	92.6% (187/202)	88–95.8	6.73	0.54
>8	42.7% (29/68)	30.7–55.2	98% (198/202)	95–99.5	21.54	0.59
>9	23.5% (16/68	14.1–35.4	99.5% (201/202)	97.3–100	47.53	0.77
>10	10.3% (7/68)	4.2–20.1	100% (202/202)	98.2–100		0.9
>11	0% (0/68)	0–5.3	100% (202/202)	98.2–100		1

*n* number, *Ben* benign, *Mal* malignant, *Sens* sensitivity, *CI* confidence interval, *Spec* specificity, *+LR* positive likelihood ratio, −LR negative likelihood ratio, *∑* sum

**Table 6:** Diagnostic parameters and cut-off values of the *Tree* flowchart in non-mass lesions.

Non-mass lesions						
Cut-off	Sens	95% CI	Spec	95% CI	+LR	-LR
≥1	100% (30/30)	88.4–100	0%	0–2.2	1	
>2	100% (30/30)	88.4–100	2.37% (4/169)	0.6–5.9	1.02	0
>3	66.67% (20/30)	47.2–82.7	87.57% (148/169)	81.6–92.1	5.37	0.38
>5	30% (9/30)	14.7–49.4	97.63% (165/169)	94.1–99.4	12.67	0.72
>7	20% (6/30)	7.7–38.6	98.22% (166/169)	94.9–99.6	11.27	0.81
>8	3.33% (1/30)	0.1–17.2	100% (169/169)	97.8–100		0.97
>9	0% (0/30)	0–11.6	100% (169/169)	97.8–100		1

*n* number, *Ben* benign, *Mal* malignant, *Sens* sensitivity, *CI* confidence interval, *Spec* specificity, *+LR* positive likelihood ratio, −LR negative likelihood ratio, *∑* sum

Of 371 benign lesions, 103 (27.8%) (99 mass and four non-mass lesions) could have been predicted using the *Tree* flowchart, with a cut-off of ≤2 in mass and non-mass lesions ruling out malignancy, without gaining any false-negative results (NPV = 100%). A cut-off of ≤2 means that all lesions, but without the root sign and with persistent or plateau enhancement regardless of the lesion margins (smooth or irregular), would be considered benign.

A cut-off of >8 in the *Tree* flowchart to rule in malignancy would have predicted 30 of 98 true-positive findings (30.6%), with a specificity of 98.9% and a positive likelihood ratio of 28.4 (Table 4). A cut-off of >8 includes all lesions showing washout with or without the root sign and lesions with plateau enhancement and the root sign.

### Inter-reader agreement

Two readers independently read 82 consecutive cases. The kappa agreement among the two readers for the characterization of breast lesions according to the *Tree* flowchart was almost perfect (k =0.944 [95% CI: 0.889–0.998]).

## Discussion

Our study shows that using a simple classification system (the *Tree* flowchart), malignancy can be excluded in 27.8% of MRI-only lesions previously classified as BI-RADS 4, without resulting in any false-negative findings, thus leading to a substantial decrease in unnecessary biopsies. Therefore, the *Tree* flowchart holds the potential to reduce the number of costly and time-consuming MRI-guided biopsies, and therefore to decrease healthcare costs, patient discomfort and the risk of possible adverse effects due to the invasive procedure. The *Tree* flowchart is intuitive and suitable for readers of different levels of experience, and it is easily applicable in the routine clinical setting, as it requires standard breast MRI sequences only.

As the *Tree* flowchart is based on T2-weighted sequences and dynamic, contrast-enhanced, T1-weighted sequences that are generally recommended for every breast MRI, it does not require any additional imaging [2]. Of note, short tau inversion recovery (STIR) sequences are as suitable as T2-weighted TSE sequences for the assessment of oedema [28]. Furthermore, the *Tree* flowchart is based on simple dynamic and morphological features. Thus, its application does not require any extra reading or computational time, as the additional acquisition of DWI would, for example [30, 31].

To date, lesion analysis based on the *Tree* flowchart does not include additional functional imaging, such as DWI or MR spectroscopy. Several studies have shown that DWI, as an adjunct to conventional breast MRI, holds the potential to reduce false-positive results and increase specificity with lower ADC values exhibited by malignant versus benign lesions [32–35]. In an analogous fashion, DWI may be combined with the *Tree* flowchart. Considering the current trend of combining information from multiple sources, referred to as *Big Data*, it is conceivable that more complex computer-assisted algorithms will be developed that rely not only on semantic and agnostic imaging features, but also on clinical background information as well as follow-up examinations. However, there are limitations concerning artifacts and spatial distortions, and different DWI approaches have prevented the determination of a generalizable ADC threshold value to distinguish malignant from benign lesions [34, 36–38].

The signs included in the *Tree* flowchart (root sign, enhancement kinetics, lesion margin, internal enhancement pattern and ipsilateral oedema) were selected initially based on their non-redundancy and the association with malignant breast lesions [23, 25–27]. More recent studies have proven the reliability and significance of these signs [17, 24, 39–41]. The *Tree* flowchart was initially evaluated in an exploratory study [23], and thereafter validated in a single clinical centre [24]. In this study, the *Tree* flowchart was applied to all patients undergoing MRI-guided VABBs in a large tertiary breast-care centre, where patients present with imaging acquired either at the same centre or at other institutions for a second reading of their MRI. This setting allows the application of the *Tree* flowchart to images from different clinical centres. After the initial two studies on homogeneous patient cohorts, this study set out to test the practical application of this decision algorithm. In the daily practice of an assessment centre, breast radiologists need to deal with imaging of varying quality. This is a setting where the *Tree* flowchart can show its true potential to reduce unnecessary breast biopsies.

This study aimed to establish the diagnostic value of the *Tree* flowchart in suspicious lesions visible only on MRI in order to provide clinical guidance in this setting. Rather than the mere application of a single cut-off value at the left upper part of the ROC curve, which maximizes the sum of sensitivity and specificity, different cut-off values can be applied according to the population in question to rule-out or rule-in malignancy [42]. Marino et al. recommended a cut-off value of ≤4 to rule-out malignancy in their cohort [24]. We identified a non-negligible number of malignant lesions in *Tree* scores 3 and 4 in our cohort of MRI-only lesions (false negative score 3 lesions comprised 9 DCIS, 8 IDC and one ILC and the only false negative score 4 lesion measured 4 mm on the MRI images and histopathology revealed a 1.5 mm invasive mucinous carcinoma). Although the number of invasive cancers in this group was very small at 4.5% (ten of 222), a cut-off of ≤2 might be preferable in the investigated setting to achieve the highest possible sensitivity. In this respect, score 4 deserves further research as we only identified one malignant lesion in this small subgroup.

Both the MRI BI-RADS terminology and the *Tree* flowchart show higher diagnostic accuracy in mass lesions than in non-mass lesions [43–46]. In MRI-only mass lesions, the *Tree* flowchart improved diagnostic accuracy by correctly identifying 99 benign lesions out of 270 lesions previously classified as BI-RADS 4 (33.3%) and four benign lesions out of 199 non-mass lesions (2.0%).

This study shows that the *Tree* flowchart can guide decision-making after discrepant results between MRI-guided biopsy and imaging. A relevant rate of false-negative histopathological results are initially obtained by MRI-guided biopsy of suspicious breast lesions visible on MRI (0–17%) [6, 22, 47–49]. In these cases, potential radiological-pathological mismatch needs to be assessed. With a test that would provide high specificity, such as the *Tree* flowchart, the radiologist may confidently request surgical biopsy after a negative histopathological result [9, 22]. This is particularly the case for lesions assigned a *Tree* result of >8, resulting in a positive likelihood ratio of >28, and thus practically ruling in malignancy. A negative biopsy result in such a case should immediately be referred either to repeat-biopsy or open surgery.

Two radiologists with different experience in breast MRI evaluated lesions according to the *Tree* flowchart with almost perfect inter-reader agreement, demonstrating that the *Tree* flowchart is easily applicable.

The primary limitation of this study was that the standard MRI scans analysed in this study were acquired on different scanner types at different field strengths and with different sequence parameters. On the one hand, the different image qualities and imaging protocols may have limited imaging interpretation based on the *Tree* flowchart and may be one reason why the rate of malignancies in flowchart nodes 3 and 4 was found to be somewhat higher than that previously reported [24]. On the other hand, the inclusion of MRI scans of different image qualities shows the reliability of the *Tree* flowchart in a realistic and heterogeneous setting of MR images, as may be seen at any reference centre offering MRI-guided VABB to patients referred from other institutions. The high inter-reader agreement and the diagnostic accuracy reported in this setting for both mass and non-mass lesions prove the applicability of the *Tree* flowchart in this heterogeneous cohort. In addition, subgroup analysis did not show a significant influence of MRI vendor on the accuracy of the *Tree* flowchart, thus corroborating its robustness regarding imaging protocol variations. Furthermore, only histopathologically verified MRI-only lesions were included in this study. Thus, the results may not apply to a general population or to all lesions visible on MRI in a similar manner. Further studies will be necessary to evaluate the applicability of the *Tree* flowchart to different subpopulations.

In conclusion, this study showed that the *Tree* flowchart, with a cut-off value of ≤2, can reduce the number of biopsies in MRI-only lesions by as much as 27.8%, with no false-negative cases, thus potentially decreasing healthcare costs and patient discomfort.

## Electronic supplementary material

Below is the link to the electronic supplementary material.

ESM 1 (DOCX 20 kb)

## Abbreviations

-LR: Negative likelihood ratio
+LR: Positive likelihood ratio
ACR BI-RADS: American College of Radiology Breast Imaging and Reporting Data System
ADC: Apparent diffusion coefficient
AUC: Area under the curve
ben: Benign
CI: Confidence interval
DCIS: Ductal carcinoma in situ
DWI: Diffusion-weighted imaging
EUSOMA: European Society of Breast Cancer Specialists
FA: Flip angle
FFE: Fast field echo
FLASH: Fast low angle shot
FOV: Field of view
FS: Fat saturation
high-risk: High-risk lesion
mal: Malignant
mm: Millimetre
MRI: Magnetic resonance imaging
ms: Millisecond
n: Number
NPV: Negative predictive value
ROC: Receiver operating characteristic
∑: SumSD, Standard deviation
sens: Sensitivity
SPAIR: Spectral attenuated inversion recovery
spec: Specificity
ST: Slice thickness
STIR: Short tau inversion recovery
T: Tesla
TE: Echo time
TI: Inversion time
TIRM: Turbo inversion recovery magnitude
TR: Repetition time
VABB: Vacuum-assisted breast biopsy
