# Supplementary material for: A simple classification system (the Tree flowchart) for breast MRI can reduce the number of unnecessary biopsies in MRI-only lesions
Source: Eur Radiol. 2017 Mar 8;27(9):3799–809. doi: 10.1007/s00330-017-4755-6 (PMC5544808; doi:10.1007/s00330-017-4755-6)
Supplement: Supplementary file 1 — (DOCX 20 kb) [file 330_2017_4755_MOESM1_ESM.docx]

| **sequence** | **Tesla** | **orientation** | **patients** | **TE [ms]** | **TR [ms]** | **TI [ms]** | **FA [°]** | **ST [mm]** | **gap [mm]** | **matrix** | | | **FOV [mm²]** |
| --- | --- | --- | --- | --- | --- | --- | --- | --- | --- | --- | --- | --- | --- |
| T1 2D FLASH | 1.5 | axial | 46 | 4.76 | 128 | - | 80 | 3 | 3.3 | 448 | x | 314 | 350 |
| T1 3D FFE | 1.5 | axial | 13 | 4.6 | 7.9-8.9 | - | 12-20 | 2-3.5 | - | 432-448 | x | 328-385 | 224-262 |
| T1 3D FFE | 3 | axial | 4 | 2.3 | 5.67 | - | 12 | 1.8 | - | 424 |  | 424 | 282 |
| T1 3D FLASH | 1.5 | axial | 297 | 4.7-5 | 7.75-12 | - | 20-25 | 2-4 | - | 384-512 | x | 328-424 | 300-400 |
| T1 3D FLASH | 1 | axial | 4 | 4.7 | 9 | - | 25 | 2.5 | - | 384 |  | 326 | 350 |
| T1 3D FLASH | 3 | axial | 4 | 2.45 | 5.47 | - | 20 | 1.75 | - | 384 |  | 342 | 360 |
| T1 3D FLASH | 1.5 | coronal | 9 | 4.76-5 | 9-12 | - | 25 | 2.5 | - | 384-448 | x | 192-326 | 340-400 |
| T1 3D FLASH | 1 | coronal | 1 | 4.7 | 9 | - | 25 | 2.5 | - | 384 |  | 326 | 340 |
| T1 3D FLASH FS | 3 | coronal | 17 | 1.21 | 3.61 | - | 6 | 2 | - | 192 | x | 192 | 360 |
| T1 3D FLASH FS | 1.5 | axial | 4 | 1.77-1.86 | 4.56-4.78 | - | 8-10 | 1.5-2 | - | 384-512 | x | 384-512 | 300-350 |
| T1 FFE | 1.5 | axial | 55 | 2.96-4.61 | 292-414 | - | 90 | 2.5-5 | 2.5-3 | 400-512 | x | 259-424 | 281-342 |
| T2 TIRM | 1.5 | axial | 239 | 61-88 | 5130-9960 | 130-170 | 150-180 | 3-4 | 3.6-5.2 | 320-448 | x | 281-336 | 300-370 |
| T2 TIRM | 1.5 | sagittal | 141 | 56-71 | 4650-4940 | 170 | 150-180 | 4 | 4.8 | 320 | x | 256 | 340-350 |
| T2 TIRM | 3 | axial | 25 | 54-65 | 3700-4800 | 230-240 | 80-120 | 3.2-4.5 | 3.2-4.5 | 320-384 | x | 204-384 | 340-360 |
| T2 STIR | 1.5 | axial | 23 | 60-70 | 1400-7812 | 160-165 | 90 | 3-4 | 3.3-4.8 | 256-352 | x | 200-280 | 340-370 |
| T2 TSE | 1.5 | axial | 13 | 69-183 | 3000-8250 | - | 160-180 | 3-5 | 1.25-4.8 | 320-596 | x | 252-592 | 320-370 |
| T2 TIRM | 1 | axial | 5 | 74 | 10000 | 130 | 150 | 4 | 4.8 | 448 | x | 336 | 300 |
| T2 SPAIR | 1.5 | axial | 8 | 173 | 1200 | - | 120 | 2 | - | 512 | x | 512 | 380 |

**Supplementary Table** Technical parameters of the evaluated T1- and T2-weighted sequences. TE = echo time, ms = milliseconds, TR = repetition time, TI = inversion time, FA = flip angle, ST = slice thickness, mm = millimeters, FOV = field of view, FLASH = fast low angle shot, FFE = fast field echo, FS = fat saturation, TIRM = turbo inversion recovery magnitude, STIR = short tau inversion recovery, SPAIR = spectral attenuated inversion recovery.
